# Sequence-Based Protein Design: A Review of Using Statistical Models to Characterize Coevolutionary Traits for Developing Hybrid Proteins as Genetic Sensors

**DOI:** 10.3390/ijms25158320

**Published:** 2024-07-30

**Authors:** Sahaj Kinshuk, Lin Li, Brian Meckes, Clement T. Y. Chan

**Affiliations:** 1 Department of Biomedical Engineering, College of Engineering, University of North Texas, 3940 N Elm Street, Denton, TX 76207, USA; sahajkinshuk@my.unt.edu (S.K.); lin.li@unt.edu (L.L.); brian.meckes@unt.edu (B.M.); 2 BioDiscovery Institute, University of North Texas, 1155 Union Circle #305220, Denton, TX 76203, USA

**Keywords:** statistical model, coevolutionary analysis, transcriptional regulator, protein design

## Abstract

Statistical analyses of homologous protein sequences can identify amino acid residue positions that co-evolve to generate family members with different properties. Based on the hypothesis that the coevolution of residue positions is necessary for maintaining protein structure, coevolutionary traits revealed by statistical models provide insight into residue–residue interactions that are important for understanding protein mechanisms at the molecular level. With the rapid expansion of genome sequencing databases that facilitate statistical analyses, this sequence-based approach has been used to study a broad range of protein families. An emerging application of this approach is to design hybrid transcriptional regulators as modular genetic sensors for novel wiring between input signals and genetic elements to control outputs. Among many allosterically regulated regulator families, the members contain structurally conserved and functionally independent protein domains, including a DNA-binding module (DBM) for interacting with a specific genetic element and a ligand-binding module (LBM) for sensing an input signal. By hybridizing a DBM and an LBM from two different family members, a hybrid regulator can be created with a new combination of signal-detection and DNA-recognition properties not present in natural systems. In this review, we present recent advances in the development of hybrid regulators and their applications in cellular engineering, especially focusing on the use of statistical analyses for characterizing DBM–LBM interactions and hybrid regulator design. Based on these studies, we then discuss the current limitations and potential directions for enhancing the impact of this sequence-based design approach.

## 1. Biological and Structural Properties of Allosterically Regulated Transcriptional Regulators

Allosterically regulated transcriptional regulators are a group of proteins that can interact with genetic elements, such as promoters, to control the expression of genes in response to specific physical and chemical stimuli. Under different conditions, the gene expression control facilitates the cells’ adaptation to environmental and physiological changes by activating desirable biological functions, including drug resistance, catabolic pathways, antibiotic biosynthesis, osmotic stress response, and bacterial pathogenicity [1,2,3,4]. Over 50 protein families of transcriptional regulators have been identified, sensing more than 200 signals [5,6]. Many regulators are characterized by a highly conserved helix–turn–helix (HTH) motif or winged helix motif. Among members in each regulator family, conserved three-dimensional structures along the helix–turn–helix or winged helix suggest that this region plays a significant structural role, which is key in their biological functions and transcriptional regulation activities. Indeed, these two motifs are the key components for binding to DNA. With the current knowledge and bioinformatics tools, potential DNA-binding proteins and regulators have often been identified based on the presence of these motifs [7,8]; thus, HTH and the winged helix are considered the core part of the DNA-binding module (DBM) among transcriptional regulators [9]. The other module, often called the ligand-binding module (LBM), is relatively less conserved, with unique sequences that may play a large role in the family members’ ability to bind and respond to different ligands [10,11].

The DBM and LBM of a regulator define DNA recognition and signal detection properties, respectively. Taking the LacI superfamily as an example, the representative family member, LacI, is composed of four monomers that form a tetramer connected by four α-helices [12,13], where each monomer contains an N-terminal DBM and an LBM towards the C-terminal. These LBMs are involved in binding the ligand as well as controlling dimerization [12,14]. Regulators in this family share a similar molecular mechanism, with the effector molecule binding to the LBM, causing a conformational change in the regulator and leading to release from the operator. In the case of the LacI protein, for which the LacI family is named, each monomer is capable of binding allolactose or IPTG; upon binding, a series of conformational changes occurs that leads to the protein releasing from the DNA, or losing the capacity to bind DNA if not bound [12].

Many regulators are essential for maintaining cell fitness in response to stresses and other biological conditions. LacI is involved in lactose metabolism by regulating the expression of the β-galactosidases lactose permease and galactoside transacetylase [15,16]. Ligand binding to the repressor causes the hinge helix to undergo a conformational change, which in turn causes changes in the helix–turn–helix motif to reduce DNA binding affinity [12], allowing access to the operon. This regulation is important, because efficient energy metabolism is a critical process for cell viability. LacI is particularly advantageous for use in genetic circuits due to the fine control that can be achieved with its promoters, largely in part due to the strong affinity of LacI for its operator *lacO*, as well as a highly dynamic range of gene expression and rapid state-switching in the presence of the inducer IPTG [17]. A range of transcriptional regulators that are mechanistically similar to LacI, such as TetR and AraC, has been used for genetic circuit construction and induction of gene expression [18,19,20,21].

## 2. Previous Development of Hybrid Regulators

**Figure 1:**
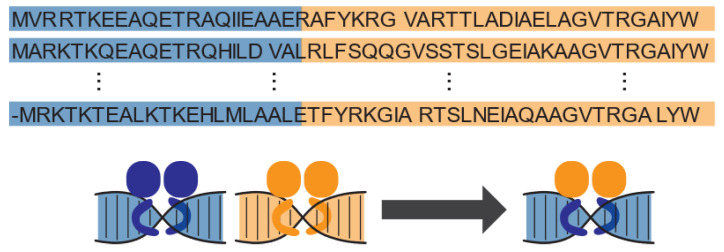
Illustration of the domain-swapping strategy. A hybrid repressor containing the DNA-binding domain from one native repressor (blue) and the ligand-binding domain of another repressor (orange) retains each native repressor’s respective DNA and ligand-binding capacities.

It has been a long-term goal in synthetic biology to utilize signal transduction pathways from different organisms to serve new purposes. These pathways are capable of sensing a broad range of conceivable bacteria-relevant inputs and linking each of them to outputs in the form of biological process regulation or more generally, gene expression [22,23]. Directly porting native sensors between organisms can be difficult, due to issues such as transcriptional incompatibilities or cross-regulation from other pathways [24]. To overcome these limitations, researchers have explored engineering transcriptional regulators with novel DNA recognition and signal detection functions, such that desirable genetic elements (such as promoters) can be chosen specifically for building a genetic network to ensure robust performance while linking the ideal signal to an output of interest. Domain swapping is one viable strategy to this end (Figure 1). Transcriptional regulators may recognize different sequences of DNA and respond to different signals, but if they are in the same protein family, meaning that they evolved from the same origin, they are structurally and mechanistically conserved. As a result, the DNA-binding modules and ligand-binding modules from different family members maintain the conserved intra-protein scaffold at the DBM–LBM interface, allowing amino acid residues across this module–module interface to interact desirably. Therefore, if DBMs and LBMs are swapped to create hybrid regulators, even if the two modules originated from different regulators, they may still be compatible for maintaining native protein structure and allosteric interactions, generating regulatory activities [9,25].

LacI is one of the first protein families to have been explored for domain swapping [26], which revealed that regulators in this family possess independent DNA-binding and regulatory domains, a feature that has been thoroughly exploited to produce hybrid regulators. Between the HTH and the ligand-binding domain, there is a linker region known as the hinge helix, and this interacts closely with the HTH to facilitate DNA-binding [14]. A change in the linker region may affect DNA-binding specificity [12,25,26]. With the well-characterized LacI family structural architecture and biochemical properties of each domain and motif, domain swapping has been robustly performed using the HTH and hinge helix as the DBM and the rest of the protein as the LBM [27,28,29].

Success in domain swapping with the LacI family has opened a new protein design strategy for developing genetic sensors. Following the initial proof of concept, researchers have demonstrated the use of this strategy in the TetR [30], LuxR [31], OmpR, NarL [32], and MerR [33] families of regulators. Taken together, this success demonstrates that domain swapping has a broad application. However, a key challenge arose; separate domains were expected to retain their functions during recombination, but in practice, domains may be incompatible due to functional or structural modifications. In many of the abovementioned studies, some of the resulting hybrid regulators possessed poor regulatory activities, which hindered their use for cellular engineering.

## 3. Genetic Circuit Development with Hybrid Regulators

Although identifying functional hybrid regulators remains challenging, some of those that have been identified have been utilized for advanced applications, such as emulating logic gate behavior at a cellular level [34,35]. The general strategy for these applications stems from the notion that regulators can be conceptualized as Boolean devices that take an input in the form of a signal ligand and produce an output such as gene expression. For example, if two repressors had the same DBM but different LBMs, then both ligands would be required to induce the expression of the common promoter, creating a genetic AND gate. In turn, linking together combinations of these components allows the design of sophisticated genetic circuits.

One of these circuits was built using hybrid repressors from the LacI family in a study by Chan et al. (2016), where a “Passcode” circuit was designed as a biocontainment system. Mediated by an AND gate of hybrid repressors containing the same DBM, two inputs were required to prevent repression of the survival signal, without which cell death was induced [36]. Another set of circuits utilizing hybrid repressors was made by Shis et al. [34], in which several highly functional (>5-fold) two-input AND gates were constructed, as well as a three-input and four-input gate. These studies highlight the novel circuit designs only attainable by using hybrid regulators. These studies primarily used LacI, possibly due to the high degree of characterization already performed by the scientific community. Later studies sought to apply the same strategy to more diverse protein families. As an example, several chimeras were constructed from the OmpR/PhoB and NarL/FixJ two-component system subfamilies using domain swapping, which were used to identify the unknown inputs to an uncharacterized two-component system [32]. Another novel application involved the use of hybrid regulators from the LuxR protein family to rewire a quorum-sensing system for controlling an endogenous biosynthetic pathway [31]. Lastly, MerR, a family of metal-responsive regulators, was used to synthesize hybrids to make circuits capable of detecting heavy metals [33].

Compared with those using only native regulators, circuits constructed with hybrid regulators that make novel connections between inputs and outputs immensely expand the range of usable signals and enable more sophisticated, multi-input circuit designs. These new connections can create circuit designs for entirely new functions that were previously impossible. However, these circuits have their own challenges, specifically in finding compatible DBMs and LBMs to connect desired inputs to outputs. When a regulator is evolving to gain new DNA recognition or signal-detection functions, it may be necessary to alter key residues involved in DBM–LBM interactions; to maintain these interactions, interacting partners may co-evolve and thus, different family members may possess a different pair of residues for a critical DBM–LBM interaction. In hybrid regulators, residues from different native regulators may not interact efficiently, leading to incompatibility between some modules. To determine module compatibility, the strategy often used is to systematically test every combination experimentally; however, this wastes significant time and resources as many nonfunctional regulators must be explored until an effective one is found. To reduce this cost of development, a relatively new approach is to use predictive models based on coevolution to identify functional regulators without needing to synthesize each one and test for efficacy until one works. Based on the pattern of amino acid distribution among family members, co-evolving residue pairs can be identified, which provides a means to determine module compatibility and changes in residues that restore perturbed interactions. Implementation of this strategy is described in Section 4, Section 5, Section 6 and Section 7.

## 4. Using Direct Coupling Analysis and Direct Information Methods to Understand Coevolution among Proteins

Statistical models have been used to analyze a wide range of proteins to reveal coevolutionary information, facilitating the understanding of these macromolecules and their interactions. A statistical modelling strategy has been used to predict performance of hybrid regulators [28,37], involving statistical methods, direct coupling analysis (DCA), and direct information (DI) [38,39]. DCA itself simply quantifies the relationship between residue positions; however, this relationship strength can be interpreted as the result of coevolutionary pressure [40]. Studies have demonstrated the capacity of these methods to generate protein structure predictions from the sequence information alone [41]. Other studies have employed DCA to augment the approach using additional methods or experimental data, such as coevolution alongside mass spectrometry data for improved determination of protein folding [42]. Additionally, DCA has been used to study protein stability and structure, as a basis for inferring contact between residues [43], and has been further integrated with molecular dynamics simulations and empirical force-field calculations for predicting protein folding [44]. Furthermore, an approach using DCA and coupled DI pairs was used to understand and model dimerization in bacterial flagellar motors [45]. Not merely limited to protein structures, DCA has been used to predict 3D RNA structures [46]. Lastly, DCA has also been extensively used to study interactions between proteins, such as between ribosome and trp operon proteins [47], as well as large-scale networks spanning entire protein families [48,49]. Variants of the above models have also been shown to be effective for protein structure prediction. A modified direct information score (DIS) based on the DI method has advanced our understanding of how bacterial two-component signaling proteins selectively interact with their appropriate partners while avoiding non-partners [50]. Additionally, pseudo-likelihood maximization-based DCA (plmDCA) and Boltzmann–machine learning-based DCA (bmDCA) [51], both derived from the original DCA model, have shown that capturing the overall statistical properties of a protein family can identify correlations between amino acid pairs in sequence alignments. This capability is crucial for accounting for protein folding and function [52].

A key advantage of DCA and the statistical model-based protein design strategy is that the only required inputs are protein sequences. Other protein design strategies have also generated great success in understanding protein mechanisms and advancing protein design, such as methods that involve calculations of free-energy states [53,54], protein motion [55,56], and the number and duration of recurrences in dynamic protein systems [57,58], which have been used to predict folding and interactions. Comparatively, these other approaches can provide more in-depth analyses of biophysical aspects, but they have a greater demand for computational power and information on protein structure. DCA is an appropriate approach for studying protein families that are not well-characterized but have a large set of available member sequences.

## 5. Using Statistical Modelling of Coevolution to Reveal Key DBM–LBM Interactions

**Figure 2:**
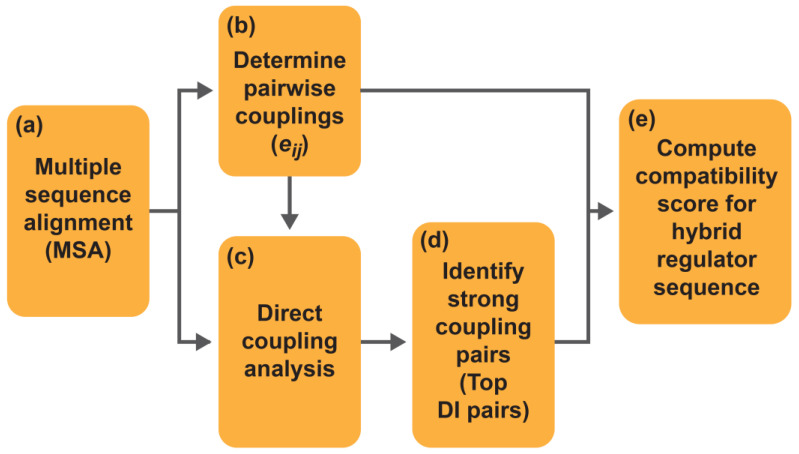
A general scheme for developing predictive models for DBM–LBM compatibility among transcriptional regulators. (**a**) Multiple sequence alignment is created using a seed sequence, which is usually from a well-characterized regulator in the protein family. (**b**) Pairwise couplings (*e_ij_*) are determined for each combination of amino acids in each residue pair between DBM and LBM. (**c**) Pairwise couplings are inputs for direct coupling analysis, which outputs direct information (DI) values for each DBM–LBM pair among family members; DI provides a quantitative means to suggest the likelihood of the two corresponding positions co-evolving. (**d**) The top DI pairs are then selected for use in model development, alongside the pairwise couplings to (**e**) generate compatibility predictions for a given set of aligned hybrid sequences.

As described above, DCA and DI have been used for studying many aspects of protein sciences. A novel application is to predict compatibility between DBMs and LBMs (Figure 2) [28]. In this strategy, functional regulators are still validated experimentally, but the time and resource costs can be significantly reduced. This model is enabled by the theory that residues on different positions of a protein that interact closely, such as to coordinate allosteric regulation, will reciprocally affect each other’s evolution. In other words, changing the residue at one site will be matched with a change of its interacting partner at another site [38]. In examples where this strategy was first applied, DCA was first used to discern residue pairs that were directly correlated due to roles in structure or function. DCA was able to identify directly correlated residue pairs from false positives caused by background signals or phylogenetic linkage. The DCA algorithm begins with a multiple-sequence alignment (MSA), generated using hidden Markov models (HMMs). MSA is critical as this step defines positional alignment for all sequences, which is the basis for statistical analysis. There are many MSA programs [59,60,61,62]; for example, HMMER [63] was used for MSA to develop hybrid regulators from the LacI family [37]. MSA starts with a sequence homology search, where a seed sequence is compared to a large database of known sequences, and known related sequences are found using statistical methods. Ideally, a large number of related sequences are found, and these sequences are used to generate a profile HMM, a hidden Markov model that is used to create a scoring system that relates the amino acid probability distribution at each position [64] and is used to detect distant homology. This method can better recognize the biases at each sequence position towards different amino acids. Additionally, profile HMMs can better detect homologs when insertions, deletions, and substitutions are involved [65]. After the profile is created, it is compared against a sequence database to identify more related sequences, after which each sequence is aligned to the profile HMM, and the final MSA is output as a single file of aligned sequences. The quality of the MSA is critical because each subsequent step is performed using the MSA as the primary input. If the MSA is constructed poorly, the downstream statistical methods yield poor results.

Following MSA generation, DCA is performed to assess each aligned residue position in the MSA *i*, and the algorithm keeps a frequency count for each amino acid *A* in column *i*. Another frequency count tracks when each amino acid *B* coappears in another residue position *j* in the same protein sequence. These coappearing frequencies are adjusted in the statistical model to avoid biases towards highly abundant genes; in a protein sequence database, regulator sequences gathered from different organisms can be nearly identical as some regulators are highly abundant across a broad spectrum of organisms. To correct for sampling bias, sequences with greater than 80% identity are counted and reweighted. With all these considerations, the equation for the statistical model *P*(*S*) of the amino acid distribution is defined as:

(1) $$P\left(S\right)=\frac{1}{Z}\left\{exp\left\{\sum_{i<j}e_{ij}\left(A_{i}A_{j}\right)+\sum_{i}h_{i}\left(A_{i}\right)\right\}\right\}$$

Here, the model is based on the maximum entropy principle [38], which attempts to make the fewest assumptions and selects the distribution with the highest entropy. This leads to a Boltzmann distribution where the energy expression is substituted for the sum of pairwise couplings *e_ij_*(*A*,*B*) and local biases *h_i_*(*A*,*B*). *Z* is the normalization factor, also called the partition function, which involves a sum of every possible state of the system, specifically *q^L^* terms, where *q* represents the 21 possible elements, 20 amino acids or a gap, and *L* is the length of the protein sequence. Due to the limits of reasonable computing power, approximations are made to reduce these terms. With this information on amino acid distribution, the direct information (DI) method can be used to determine co-evolving pairs of residues, as the next step for developing the predictive model for hybrid regulator design.

**Figure 3:**
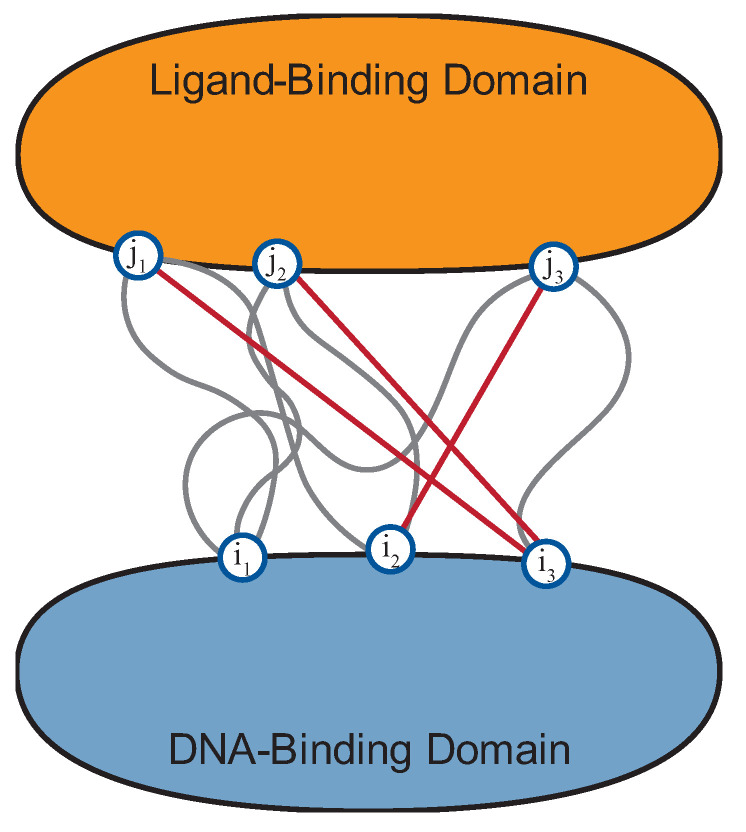
Identification of top coupling residue position pairs informs regulator design by highlighting key interactions that may be disrupted following domain swapping. In the illustration, it is implied that a hybrid regulator’s poor function may be due to lost interactions between positions *j*_1_, *j*_2_, and *i*_3_.

To understand coupling strengths between pairs of specific residue positions, direct information is computed, representing the inference of direct statistical couplings, resulting in a quantification of correlation strength between columns in the MSA [38]. Direct information is described via the following equation [28]:

(2) $${DI}_{ij}=\sum_{A_{i}A_{j}}P_{ij}\left(A_{i},A_{j}\right)ln\frac{P_{ij}\left(A_{i},A_{j}\right)}{f_{i}\left(A_{i}\right)f_{j}\left(A_{j}\right)}$$

where the probability distribution *P_ij_*(*A_i_*, *A_j_*) describes the variables that are coupled by the direct link. In this expression, *f_i_* and *f_j_* are the correct marginal distributions for the interaction. This quantity can be measured for every combination of positions *i* and *j*. Sorting this list from the greatest DI value to the lowest provides an ordered list of statistically inferred strongest coupling residue positions, which can be used to inform hybrid regulator design, as illustrated in Figure 3. In the study performed on LacI family regulators, the top 1500 DBM–LBM interaction pairs from the total possible 14,711 DBM–LBM pairs were selected to exclude noise from non-co-evolving pairs [28]. These 1500 pairs were then used as inputs for module compatibility predictions.

## 6. Predictive Model Development for Evaluating DBM–LBM Compatibility

With key DBM–LBM interaction pairs identified from DCA and DI, these pairs were used to develop a model for predicting compatibility between DBM and LBM in hybrid regulators. The principle of this predictive model is to compare the patterns of amino acid residue compositions in these pairs between a regulator sequence and the statistical trend in the entire family; when a regulator sequence has a residue composition pattern similar to that of the majority of the population, it is expected that these DBM–LBM pairs can maintain desirable interactions and thus, the regulator should be functional.

To design hybrid regulators, their sequences can be systematically generated by using the DBM of one native regulator from the original MSA and the LBM of another native regulator, to create an array of length *L*^2^ − *L*, where *L* is the list of native regulators of interest. The final list is composed of each combination of DBM–LBM, excluding the original native sequences.

A compatibility score C(S) is then generated for each hybrid regulator sequence by summing the *e_ij_* values (see Equation (1)) for each amino acid pair in the sequence, as described by the top selected DI pairs, mathematically depicted with the sigma notation in Equation (3). In other words, for each DI pair in the list, the algorithm finds which two residues are in those positions in the sequence and returns the corresponding coupling value from the *e_ij_* submatrix for those two residues for that DI pair, and that value is added to the sum for that sequence. The total sum of all these coupling values is the score *C*(*S*) for that hybrid regulator sequence, which is described by the following equation:

(3) $$C\left(S\right)=\sum_{i}^{DBM}\sum_{j}^{LBM}\left\{-e_{ij}\left(A_{i}A_{j}\right)\right\}$$

For the application of hybrid regulator module compatibility prediction, position *i* is always in the DBM and position *j* is always in the LBM. The algorithm sums each coupling value for the amino acids in positions *i* and *j* for each *i*,*j* combination in the input list of DI pairs. The more negative a *C*(*S*) score, the greater the prediction that the hybrid regulator will be functional [28].

## 7. Hybrid Regulator Rescue Using the Coevolutionary Predictive Model Approach

In addition to predicting hybrid regulators’ performance, the coevolution-based model has been used to design mutants for improving protein activities. Taking the predictive model a step further, it can evaluate how point mutations may affect compatibility between DBMs and LBMs, based on the change in the compatibility score. A mutation that leads to a more favorable score can be considered constructive for facilitating key DBM–LBM interactions. Therefore, if a hybrid regulator is poorly functional due to the loss of interactions, the model can be used to guide the design of mutations for restoring those interactions, improving compatibility between the DBM and LBM. Jiang et al. used this approach to rescue the activities of a range of hybrid regulators [37]. In that example, the team generated the full set of sequences with a single mutation in the LBM for each target hybrid regulator. Then, the predictive model was used to compute their compatibility score. By experimentally characterizing the four mutations that led to the largest improvements in the score, they identified mutants that significantly improved the activities of four hybrid regulators.

Furthermore, the team used a similar statistical approach for coevolutionary analysis to evaluate the potential of mutations to generate adverse effects on protein structure and function. As an amino acid residue may interact with multiple residues [66,67,68], mutating a residue to reinstall DBM–LBM interactions may alter other critical interactions, hampering protein structure stability. To predict how mutations may affect the protein, a structural fitness model was developed, which also involved DCA to identify key intra-module interaction pairs and define the statistical patterns of residue combinations in these pairs [37]. Similar to the compatibility model *C*(*S*) (Equation (3)), these results were used to compute the structural fitness score for mutant sequences, indicating the risk of protein degradation by the mutation. This structural fitness model provided an additional layer of information for designing hybrid regulator mutants.

## 8. Limitations of the Statistical Model Approach and Its Future Development

The success in designing LacI family regulators with sequence-based global statistical analyses has provided a strong foundation in techniques and knowledge to apply this approach to other protein families. However, some barriers are expected when extending its use for hybrid regulator design. One of the main limitations is related to the size of the protein family; to accurately identify coevolutionary traits with statistical models, previous studies suggested that more than 1000 homologous, but sufficiently divergent, sequences are required [38,48]. However, many regulators have great potential for genetic-sensor applications although their families do not meet this criterion. For example, in the Pfam database, families of *Thermus thermophilus* FadR (PF21776), *Staphylococcus aureus* IcaR (PF18665), and *Mycobacteria smegmatis* DarR (PF17932) have less than 500 members. Fortunately, with the rapid expansion of the genome sequencing database, the number of regulatory sequences is expected to increase in the near future, reducing this problem.

As another limitation, statistical analyses on protein sequences can reveal only residue–residue interactions and not the interactions between residues and other biological components, such as DNA and signaling molecules. Regulatory functions involve binding to DNA at the DBM and signaling molecules at the LBM; these properties may be perturbed when designing mutants to reinstall DBM–LBM interactions. Avoiding this situation requires an in-depth understanding of the structural and biochemical properties of the regulator family, which then allows the selection of appropriate mutations. Additional computational tools can be used to determine interactions between transcriptional regulators and other biological molecules. Recent advances in computational biology support accurate predictions of protein–ligand [69] and protein–nucleic acid [70] interactions. Additionally, breakthroughs in artificial intelligence-based technologies facilitate robust characterization of interactions among biological molecules [71]. The limitations can potentially be overcome with these complementing technologies.

Additionally, the use of this approach for protein design can be facilitated by developing comprehensive software with a user-friendly interface to automate all the steps in the model development, ranging from multiple sequence alignment to score computing. This would eliminate the barrier for researchers without a computational background to gain access to these techniques. Studies from the previous several years demonstrate a promising pathway for the robust design of hybrid regulators, and by addressing these abovementioned issues, the statistic model approach will be broadly applied to study other regulator families, as well as other multidomain proteins that involve conserved domain–domain interactions.

## 9. Conclusions

The development of hybrid transcriptional regulators represents a way to create biomolecular parts for synthetic gene networks, in which these new parts are the key to new circuit designs. Recent studies on LacI family hybrid regulators show a promising path to extend the capabilities in designing hybrid regulators by using a statistical model approach for coevolutionary analysis. Resulting coevolutionary cues not only provide a means to predict compatibility between DBMs and LBMs, they also support the design of mutations that restore DBM–LBM interactions for rescuing regulator activities. These previous studies have established a strong foundation for exploring this approach to design hybrid regulators in other protein families.

## References

[1] Ramos J.L., Martinez-Bueno M., Molina-Henares A.J., Teran W., Watanabe K., Zhang X., Gallegos M.T., Brennan R., Tobes R. (2005). The TetR family of transcriptional repressors. Microbiol. Mol. Biol. Rev..

[2] Romero-Rodriguez A., Robledo-Casados I., Sanchez S. (2015). An overview on transcriptional regulators in Streptomyces. Biochim. Biophys. Acta.

[3] Baugh A.C., Momany C., Neidle E.L. (2023). Versatility and Complexity: Common and Uncommon Facets of LysR-Type Transcriptional Regulators. Annu. Rev. Microbiol..

[4] Cuthbertson L., Nodwell J.R. (2013). The TetR family of regulators. Microbiol. Mol. Biol. Rev..

[5] Novichkov P.S., Kazakov A.E., Ravcheev D.A., Leyn S.A., Kovaleva G.Y., Sutormin R.A., Kazanov M.D., Riehl W., Arkin A.P., Dubchak I. (2013). RegPrecise 3.0—A resource for genome-scale exploration of transcriptional regulation in bacteria. BMC Genom..

[6] Mistry J., Chuguransky S., Williams L., Qureshi M., Salazar G.A., Sonnhammer E.L.L., Tosatto S.C.E., Paladin L., Raj S., Richardson L.J. (2021). Pfam: The protein families database in 2021. Nucleic Acids Res..

[7] Huffman J.L., Brennan R.G. (2002). Prokaryotic transcription regulators: More than just the helix-turn-helix motif. Curr. Opin. Struct. Biol..

[8] Yu Z., Reichheld S.E., Savchenko A., Parkinson J., Davidson A.R. (2010). A comprehensive analysis of structural and sequence conservation in the TetR family transcriptional regulators. J. Mol. Biol..

[9] Chan C.T.Y., Kennedy V., Kinshuk S. (2024). A domain swapping strategy to create modular transcriptional regulators for novel topology in genetic network. Biotechnol. Adv..

[10] Deng W., Li C., Xie J. (2013). The underling mechanism of bacterial TetR/AcrR family transcriptional repressors. Cell Signal..

[11] Herde Z.D., Short A.E., Kay V.E., Huang B.D., Realff M.J., Wilson C.J. (2020). Engineering allosteric communication. Curr. Opin. Struct. Biol..

[12] Swint-Kruse L., Matthews K.S. (2009). Allostery in the LacI/GalR family: Variations on a theme. Curr. Opin. Microbiol..

[13] Sauer R.T. (1996). Lac repressor at last. Structure.

[14] Bell C.E., Lewis M. (2000). A closer view of the conformation of the Lac repressor bound to operator. Nat. Struct. Biol..

[15] Hersey A.N., Kay V.E., Lee S., Realff M.J., Wilson C.J. (2023). Engineering allosteric transcription factors guided by the LacI topology. Cell Syst..

[16] Lewis M. (2005). The lac repressor. Comptes Rendus Biol..

[17] Saenger W., Orth P., Kisker C., Hillen W., Hinrichs W. (2000). The Tetracycline Repressor—A Paradigm for a Biological Switch. Angew. Chem. Int. Ed. Engl..

[18] Chen D., Arkin A.P. (2012). Sequestration-based bistability enables tuning of the switching boundaries and design of a latch. Mol. Syst. Biol..

[19] Tigges M., Denervaud N., Greber D., Stelling J., Fussenegger M. (2010). A synthetic low-frequency mammalian oscillator. Nucleic Acids Res..

[20] Elowitz M.B., Leibler S. (2000). A synthetic oscillatory network of transcriptional regulators. Nature.

[21] Friedland A.E., Lu T.K., Wang X., Shi D., Church G., Collins J.J. (2009). Synthetic gene networks that count. Science.

[22] Kiel C., Yus E., Serrano L. (2010). Engineering signal transduction pathways. Cell.

[23] Rowland M.A., Fontana W., Deeds E.J. (2012). Crosstalk and competition in signaling networks. Biophys. J..

[24] Vert G., Chory J. (2011). Crosstalk in cellular signaling: Background noise or the real thing?. Dev. Cell..

[25] Meinhardt S., Manley M.W., Becker N.A., Hessman J.A., Maher L.J., Swint-Kruse L. (2012). Novel insights from hybrid LacI/GalR proteins: Family-wide functional attributes and biologically significant variation in transcription repression. Nucleic Acids Res..

[26] Tungtur S., Egan S.M., Swint-Kruse L. (2007). Functional consequences of exchanging domains between LacI and PurR are mediated by the intervening linker sequence. Proteins.

[27] Groseclose T.M., Hersey A.N., Huang B.D., Realff M.J., Wilson C.J. (2021). Biological signal processing filters via engineering allosteric transcription factors. Proc. Natl. Acad. Sci. USA.

[28] Dimas R.P., Jiang X.L., Alberto de la Paz J., Morcos F., Chan C.T.Y. (2019). Engineering repressors with coevolutionary cues facilitates toggle switches with a master reset. Nucleic Acids Res..

[29] Rondon R.E., Wilson C.J. (2019). Engineering a New Class of Anti-LacI Transcription Factors with Alternate DNA Recognition. ACS Synth. Biol..

[30] Dimas R.P., Jordan B.R., Jiang X.L., Martini C., Glavy J.S., Patterson D.P., Morcos F., Chan C.T.Y. (2019). Engineering DNA recognition and allosteric response properties of TetR family proteins by using a module-swapping strategy. Nucleic Acids Res..

[31] Mukherji R., Zhang S., Chowdhury S., Stallforth P. (2020). Chimeric LuxR Transcription Factors Rewire Natural Product Regulation. Angew. Chem. Int. Ed. Engl..

[32] Schmidl S.R., Ekness F., Sofjan K., Daeffler K.N., Brink K.R., Landry B.P., Gerhardt K.P., Dyulgyarov N., Sheth R.U., Tabor J.J. (2019). Rewiring bacterial two-component systems by modular DNA-binding domain swapping. Nat. Chem. Biol..

[33] Ghataora J.S., Gebhard S., Reeksting B.J. (2023). Chimeric MerR-Family Regulators and Logic Elements for the Design of Metal Sensitive Genetic Circuits in Bacillus subtilis. ACS Synth. Biol..

[34] Shis D.L., Hussain F., Meinhardt S., Swint-Kruse L., Bennett M.R. (2014). Modular, multi-input transcriptional logic gating with orthogonal LacI/GalR family chimeras. ACS Synth. Biol..

[35] Milner P.T., Zhang Z., Herde Z.D., Vedire N.R., Zhang F., Realff M.J., Wilson C.J. (2023). Performance Prediction of Fundamental Transcriptional Programs. ACS Synth. Biol..

[36] Chan C.T., Lee J.W., Cameron D.E., Bashor C.J., Collins J.J. (2016). ‘Deadman’ and ‘Passcode’ microbial kill switches for bacterial containment. Nat. Chem. Biol..

[37] Jiang X.L., Dimas R.P., Chan C.T.Y., Morcos F. (2021). Coevolutionary methods enable robust design of modular repressors by reestablishing intra-protein interactions. Nat. Commun..

[38] Morcos F., Pagnani A., Lunt B., Bertolino A., Marks D.S., Sander C., Zecchina R., Onuchic J.N., Hwa T., Weigt M. (2011). Direct-coupling analysis of residue coevolution captures native contacts across many protein families. Proc. Natl. Acad. Sci. USA.

[39] Dinan J.C., McCormick J.W., Reynolds K.A. (2024). Engineering Proteins Using Statistical Models of Coevolutionary Sequence Information. Cold Spring Harb. Perspect. Biol..

[40] Kamisetty H., Ovchinnikov S., Baker D. (2013). Assessing the utility of coevolution-based residue-residue contact predictions in a sequence- and structure-rich era. Proc. Natl. Acad. Sci. USA.

[41] Marks D.S., Colwell L.J., Sheridan R., Hopf T.A., Pagnani A., Zecchina R., Sander C. (2011). Protein 3D structure computed from evolutionary sequence variation. PLoS ONE.

[42] Dos Santos R.N., Ferrari A.J.R., de Jesus H.C.R., Gozzo F.C., Morcos F., Martinez L. (2018). Enhancing protein fold determination by exploring the complementary information of chemical cross-linking and coevolutionary signals. Bioinformatics.

[43] Morcos F., Schafer N.P., Cheng R.R., Onuchic J.N., Wolynes P.G. (2014). Coevolutionary information, protein folding landscapes, and the thermodynamics of natural selection. Proc. Natl. Acad. Sci. USA.

[44] Sulkowska J.I., Morcos F., Weigt M., Hwa T., Onuchic J.N. (2012). Genomics-aided structure prediction. Proc. Natl. Acad. Sci. USA.

[45] Dos Santos R.N., Khan S., Morcos F. (2018). Characterization of C-ring component assembly in flagellar motors from amino acid coevolution. R. Soc. Open Sci..

[46] Weinreb C., Riesselman A.J., Ingraham J.B., Gross T., Sander C., Marks D.S. (2016). 3D RNA and Functional Interactions from Evolutionary Couplings. Cell.

[47] Feinauer C., Szurmant H., Weigt M., Pagnani A. (2016). Inter-Protein Sequence Co-Evolution Predicts Known Physical Interactions in Bacterial Ribosomes and the Trp Operon. PLoS ONE.

[48] Uguzzoni G., John Lovis S., Oteri F., Schug A., Szurmant H., Weigt M. (2017). Large-scale identification of coevolution signals across homo-oligomeric protein interfaces by direct coupling analysis. Proc. Natl. Acad. Sci. USA.

[49] Croce G., Gueudre T., Ruiz Cuevas M.V., Keidel V., Figliuzzi M., Szurmant H., Weigt M. (2019). A multi-scale coevolutionary approach to predict interactions between protein domains. PLoS Comput. Biol..

[50] Cheng R.R., Morcos F., Levine H., Onuchic J.N. (2014). Toward rationally redesigning bacterial two-component signaling systems using coevolutionary information. Proc. Natl. Acad. Sci. USA.

[51] Figliuzzi M., Barrat-Charlaix P., Weigt M. (2018). How Pairwise Coevolutionary Models Capture the Collective Residue Variability in Proteins?. Mol. Biol. Evol..

[52] Russ W.P., Figliuzzi M., Stocker C., Barrat-Charlaix P., Socolich M., Kast P., Hilvert D., Monasson R., Cocco S., Weigt M. (2020). An evolution-based model for designing chorismate mutase enzymes. Science.

[53] Leman J.K., Weitzner B.D., Lewis S.M., Adolf-Bryfogle J., Alam N., Alford R.F., Aprahamian M., Baker D., Barlow K.A., Barth P. (2020). Macromolecular modeling and design in Rosetta: Recent methods and frameworks. Nat. Methods.

[54] Simonson T., Archontis G., Karplus M. (2002). Free energy simulations come of age: Protein-ligand recognition. Acc. Chem. Res..

[55] Childers M.C., Daggett V. (2017). Insights from molecular dynamics simulations for computational protein design. Mol. Syst. Des. Eng..

[56] Rouhani M., Khodabakhsh F., Norouzian D., Cohan R.A., Valizadeh V. (2018). Molecular dynamics simulation for rational protein engineering: Present and future prospectus. J. Mol. Graph. Model..

[57] Giuliani A., Benigni R., Colafranceschi M., Chandrashekar I., Cowsik S.M. (2003). Large contact surface interactions between proteins detected by time series analysis methods: Case study on C-phycocyanins. Proteins.

[58] Bruni R., Costantino A., Tritarelli E., Marcantonio C., Ciccozzi M., Rapicetta M., El Sawaf G., Giuliani A., Ciccaglione A.R. (2009). A computational approach identifies two regions of Hepatitis C Virus E1 protein as interacting domains involved in viral fusion process. BMC Struct. Biol..

[59] Katoh K., Misawa K., Kuma K., Miyata T. (2002). MAFFT: A novel method for rapid multiple sequence alignment based on fast Fourier transform. Nucleic Acids Res..

[60] Edgar R.C. (2004). MUSCLE: A multiple sequence alignment method with reduced time and space complexity. BMC Bioinform..

[61] Notredame C., Higgins D.G., Heringa J. (2000). T-Coffee: A novel method for fast and accurate multiple sequence alignment. J. Mol. Biol..

[62] Do C.B., Mahabhashyam M.S., Brudno M., Batzoglou S. (2005). ProbCons: Probabilistic consistency-based multiple sequence alignment. Genome Res..

[63] Potter S.C., Luciani A., Eddy S.R., Park Y., Lopez R., Finn R.D. (2018). HMMER web server: 2018 update. Nucleic Acids Res..

[64] Eddy S.R. (1998). Profile hidden Markov models. Bioinformatics.

[65] Eddy S.R. (2011). Accelerated Profile HMM Searches. PLoS Comput. Biol..

[66] Gromiha M.M., Selvaraj S. (2001). Comparison between long-range interactions and contact order in determining the folding rate of two-state proteins: Application of long-range order to folding rate prediction. J. Mol. Biol..

[67] Gao J., Li Z. (2008). Inter-residue interactions in protein structures exhibit power-law behavior. Biopolymers.

[68] Vymetal J., Jakubec D., Galgonek J., Vondrasek J. (2021). Amino Acid Interactions (INTAA) web server v2.0: A single service for computation of energetics and conservation in biomolecular 3D structures. Nucleic Acids Res..

[69] Nakata S., Mori Y., Tanaka S. (2023). End-to-end protein-ligand complex structure generation with diffusion-based generative models. BMC Bioinform..

[70] Townshend R.J.L., Eismann S., Watkins A.M., Rangan R., Karelina M., Das R., Dror R.O. (2021). Geometric deep learning of RNA structure. Science.

[71] Abramson J., Adler J., Dunger J., Evans R., Green T., Pritzel A., Ronneberger O., Willmore L., Ballard A.J., Bambrick J. (2024). Accurate structure prediction of biomolecular interactions with AlphaFold 3. Nature.

